# Retrospective Analysis and Comparison of 48 Intracranial Meningioma Cases As Two Groups According to Their Size

**DOI:** 10.7759/cureus.19709

**Published:** 2021-11-18

**Authors:** Hasan Burak Gündüz, Ayşegül Esen Aydın, Seda Yağmur Karataş Okumuş, Orhun Mete Çevik, Özden Erhan Sofuoğlu, Mustafa Levent Uysal, Murad Asiltürk, Müslüm Güneş, Talat Cem Ovalıoğlu, Erhan Emel

**Affiliations:** 1 Neurosurgery, Bakirkoy Prof. Dr. Mazhar Osman Research and Training Hospital for Psychiatry, Neurology and Neurosurgery, Istanbul, TUR; 2 Neurosurgery, Bakirkoy Prof. Dr. Mazhar Osman Research and Training Hospital for Psychiatry, Neurology and Neurosurgery, istanbul, TUR

**Keywords:** karnofsky performance scale, simpson grading scale, meningioma histopathology, intracranial meningioma, giant intacranial meningioma

## Abstract

Objective

This study aims to examine the possible demographic, clinical, and surgical differences between giant and smaller meningiomas.

Materials and Methods

Forty-eight meningioma patients who were operated on in our clinic between 2016-2020 were included in our study. Fourteen meningiomas larger than 5 cm in diameter were defined as giant meningiomas and placed in group 1. Thirty-four remaining meningiomas, with sizes less than 5 cm, were placed in group 2. These patients were evaluated regarding age, sex, localization, symptoms and neurological findings, surgical results, histopathology, and postoperative results.

Results

The most common localization in group 1 was falcine-parasagittal, whereas in group 2 it was convexity. Simpson’s grade I resection rate in group 1 was 35.71%, while in group 2 this rate was 67.65%. In histopathological examination, transitional type meningiomas (35.71%) were the most common in group 1, whereas fibrous type meningiomas (32.35%) were seen the most in group 2. Group 1 Karnofsky Performance Scale score average was 75.71 preoperatively and 85.71 postoperatively. In group 2, the preoperative and postoperative average was 97.35 and 96.76, respectively. The comparative statistical analysis reflects that: A) Resection rates were significantly lower in the giant meningioma group. B) Similarly, Karnofsky Performance Scale scores were also lower than group 2. C) When statistical comparisons were made according to sex, age, localization, histopathological results, postoperative complications, and recurrence rates, no significant differences were observed.

Conclusion

The term “Giant Meningioma” is a type of distinction that is frequently made in the literature. However, the single major difference we see in our study was the surgical results. The general condition of patients before and after surgery may be more critical than others in giant meningiomas. Although surgical resection is the main form of treatment in giant meningiomas, the risks arising from the size of the tumor should be taken into account, and necessary plans should be made for a successful surgical intervention.

## Introduction

Meningiomas are usually benign and slow-growing tumors of the central nervous system. They are the second most common tumors in the central nervous system in adults. The annual incidence of meningiomas is 2.3 per 100,000. They mainly originate from arachnoid cap cells. In general, they are more common in women with a 2:1 ratio. Most meningiomas have a good prognosis [1-5]. However, since it is possible for meningiomas to be closely related to neural or vascular structures, total resection may not always be possible. In addition, since radiation therapy is limited to neurotoxicity, tumor size is one of the major factors in the remaining treatment of aggressive, inoperable, residual, or recurrent meningiomas. Regardless, tumor size is also one of the important factors directly affecting the surgical process. In many studies, meningiomas over 5-6 cm are considered under a separate category with the term “giant” or “huge” [2,3,6,7].

There are publications on the relationship between meningioma and various factors (age, location, tumor size, and WHO grade) in the literature review. The relationship between tumor size and histopathological grading was also investigated in the articles of Magill et al. and Ressel et al. [8,9]. Since “giant” meningiomas are a terminologically accepted category in the literature, it will be clinically and statistically meaningful to investigate what kind of differences this classification includes within the general definition of meningiomas.

In conclusion, our study investigates whether there is a relationship between tumor size and the independent variables of the treatment. We aim to shed light on the attributes that differentiate the meningiomas that are considered “giant” from others; or if the definition is solely dependant on the size. In this context, we evaluated and compared meningiomas, which we classified into two groups according to their dimensions, in terms of age, sex, localization, complaints, neurological findings, resection rates, histopathological classification, complications, and postoperative performance status. There is only one dependent variable as this comparison is made between tumors above and below 5cm. All other variables are based on a comparison of these criteria.

## Materials and methods

Approval for this study was obtained from the Clinical Ethics Committee of Bakirkoy Dr.Sadi Konuk Education and Research Hospital (Document number: 2020-546 Date: 18.01.2021)

Patient population

This retrospective study includes 48 patients who were operated on to diagnose an intracranial tumor in the neurosurgery clinic between January 1st, 2016, to December 31st, 2020, and whose histopathological diagnosis was concluded as meningioma. All patients were operated in the Neurosurgery Clinic of Istanbul Bakırkoy Prof. Dr. Mazhar Osman Research and Training Hospital for Neurology, Neurosurgery, and Psychiatry.

Exclusion criteria: 1) Due to ethical committee limitations, patients under 18 years of age 2) patients could not undergo surgical treatment for medical reasons.

Patients who underwent surgery were classified according to the size of their tumors based on cranial Magnetic Resonance (MR) images. Thus, patients were divided into two cohorts. Tumors with the longest dimension of 5 cm and above were included in the “giant meningiomas” (group 1), while the others were included in the” Meningiomas smaller than 5 cm” (Group 2) groups.

Demographic, clinical, radiological, and histopathological assessment

First of all, the demographic information of the patients was categorized, and both groups were compared. The complaints of patients diagnosed with meningioma during the first admission to the hospital and the neurological findings detected after the first examination were documented. These data were tabulated comparatively based on both groups. The meningiomas included in our study were classified according to their localizations, such as falcine-parasagittal, cerebral convexity, olfactory groove, tuberculum sellae, sphenoid wing, cerebellopontine angle, intraorbital and clival meningiomas. Based on the perioperative process and postoperative MR images, resection rates were graded based on the Simpson grading scale [10]. Complications observed in the postoperative period were documented. Preoperative and postoperative conditions of the patients were assessed based on the Karnofsky Performance Scale (KPS) [11,12]. Postoperative histopathological results were categorized based on the World Health Organization (WHO) 2016 classification of meningiomas [13].

All the data obtained were analyzed in two separate groups according to their dimensions. Demographic, clinical, radiological, and histopathological results were presented comparatively in tables defined based on these two groups.

Statistical assessment

The obtained results were analyzed in the PC-based SPSS statistical program (Version 16.0), and the differences between the two groups were reviewed. We compared demographic data, localization, histopathological results, resection scores according to Simpson’s scale, postoperative KPS scores, tumor sizes, complications and examined whether they were statistically significant. We applied the Shapiro-Wilk Normality test to all results. The normality test was negative in all comparisons except for age distribution. We used the Independent Samples T-test to analyze the age variable. We used Non-Parametric statistical methods for all other analyses. We used the Mann-Whitney U test to evaluate KPS score results and the Spearman test to analyze sex, localization, Simpson scoring, histopathological results, and complications.

We compared the recurrence rates of both groups with the Kaplan Meier survival analysis graph. We put the results into the logrank test and calculated the chi-square test statistics [14].

## Results

Demographic, clinical, surgical, and histopathological data are presented in the results section as comparative tables covering both groups.

Giant meningiomas (Group 1)

Of the giant meningiomas operated in our clinic, the largest was 8.55 cm, and the smallest was 5.02 cm in diameter (Table 1).

**Table 1:** Localizations, histopathologic results, and sizes of 15 giant meningiomas in 14 operated patients.

	Sex	Age	Localization	Histopathology	Simpson grading	Dimension (cm)
1	Female	56	Right olfactory groove, left frontal Falcine-parasagittal	Transitional	3	5.84x5.30x4.45 (Right) 5.07x5.25x3.71 (Left)
2	Female	56	Right sphenoid ridge	Meningothelial	1	5.99X4.29x6.24
3	Female	77	Left frontal parasagittal	Atypical	3	6.18x4.03x3.84
4	Female	58	Left parietal parasagittal	Fibrous	3	6.74x4.36x5.85
5	Female	29	Right frontal convexity	Atypical	2	8.55x5.81x7.15
6	Female	45	Left parietal convexity	Transitional	1	4.83x3.64x5.26
7	Female	34	Left parietooccipital parasagittal	Fibrous	3	4.97x4.09x5.10
8	Female	56	Left frontal convexity	Transitional	1	6.43x5.53x4.29
9	Male	85	Right frontal convexity	Transitional	1	5.82x3.57x4.77
10	Male	49	Left sphenoid ridge +orbital+cavernous sinus	Fibrous	4	5.06x4.22x4.22
11	Male	67	Right frontal parasagittal	Atypical	2	4.45x5.74x2.62
12	Female	44	Left sphenoid wing	Meningothelial	2	7.21x6.82x6.66
13	Male	58	Left clivus+pontocerebellar angle	Transitional	2	5.02x4.29x4.25
14	Male	72	Right frontal parasagittal	Transitional	1	5.03x4.56x4.90

Of the 14 patients with 5 cm and larger tumors, nine were female and five were male (1.8 / 1). The age range was 29-86 and the average age was 57.93 (SD ± 14.61). 57.14% of the cases were seen in the 4th and 5th decades (Table 2).

**Table 2:** Age and sex distribution of patients in both groups.

	Sex		Age	
	Female	Male	Average	Range
Group 1 N=14 (≥5cm)	9(64.29%)	5(35.71%)	57.93	29-86
Group 2 N=34 (<5cm)	28(82.35%)	6(17.65%)	50.23	30-71

**Figure 1:**
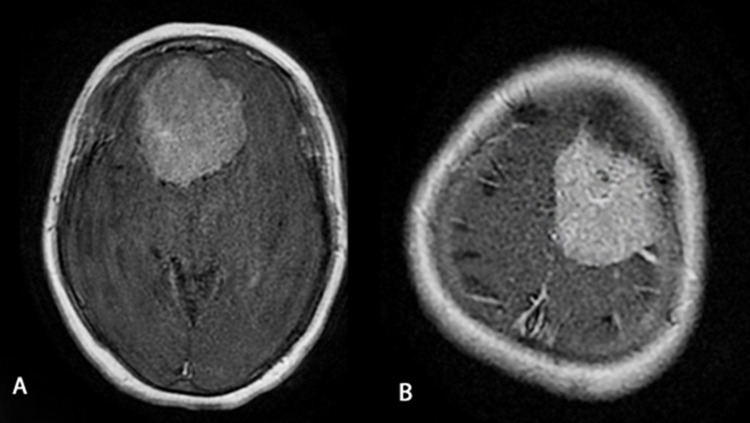
Multiple giant meningiomas. Localization: A) Right Olfactory Groove B) Left Falcine-Parasagittal.

Tumor localization was falcine-parasagittal in five patients (35.71%), convexity in four patients (28.57%), and sphenoid wing in two patients (14.29%). In addition, sphenoid wing, orbital and cavernous sinus involvement was seen in one patient, and clivus and cerebellopontine angle involvement in another patient. One of our patients had two giant tumors in different locations (Table 3 and Figure 1).

**Table 3:** Distribution of all meningiomas by localization.

Localization	≥ 5cm N=14	<5cm N=34
Falcine-parasagittal	5 (35.71%)	11 (32.35%)
Convexity	4 (28.57%)	18 (52.94%)
Sphenoid wing	2 (14.29%)	
Clivus+cerebellopontine angle	1 (7.14%)	
Sphenoid wing+ orbita+cavernous sinus	1 (7.14%)	
Multiple meningioma: Right olfactory groove +left parasagittal	1 (7.14%)	
Olfactory groove		2 (5.88%)
Suprasellar		1 (2.94%)
Tuberculum sella		1 (2.94%)
Multiple meningioma: Parietal convexity		1 (2.94%)

The most common complaint in patients with giant meningioma was headache (12-85.71%). This was followed by nausea and vomiting (6-42.86%), loss of strength (4-28.57%) and vision loss (4-28.57%). Besides, two patients had a history of seizures (14.29%), each patient had personality changes, tinnitus, swallowing difficulty, and urinary incontinence (7.14%) (Table 4).

**Table 4:** Complaints of both groups of patients at first admission.

Complaints	Group 1(≥5cm)	Group 2(<5cm)
Headache	12 (85.71%)	13(38.23%)
Nausea and vomiting	6 (42.86%)	
Loss of strength	4 (28.57%)	
Vision loss	4 (28.57%)	5(14.71%)
History of seizure	2 (14.29%)	8 (23.53%)
Personality change	1 (7.14%)	1(2.94%)
Tinnitus	1 (7.14%)	
Swallowing difficulty	1 (7.14%)	
Urinary incontinance	1 (7.14%)	
Vertigo		3(8.82%)
Dizziness		2(5.88%)
Hearing impairment		2(5.88%)
Worsening of consciousness		1(2.94%)
Facial numbness		1(2.94%)

In the neurological examination of these patients, paresis of an extremity (28.57%) was detected in four patients, decreased visual acuity in four patients (28.57%), facial paresis in two patients (14.29%), anosmia in one patient (7.14%), and ataxic walking in one patient (7.14%). Neurological examination of four patients was evaluated as natural (Table 5).

**Table 5:** Neurological findings when patients applied to the hospital.

Neurological findings	Group 1 (≥5cm)	Group 2(<5cm)
Paresis of extremity	4(%28.57)	1(%2.94)
Decreased visual acuity	4(%28.57)	6(%17.65)
Fascial paresis	2(%14.29)	1(%2.94)
Anosmia	1(%7.14)	
Ataxic walking	1(%7.14)	
Decrease in hearing		1(%2.94)
Worsening in the level of consciousness		1(%2.94)

**Figure 2:**
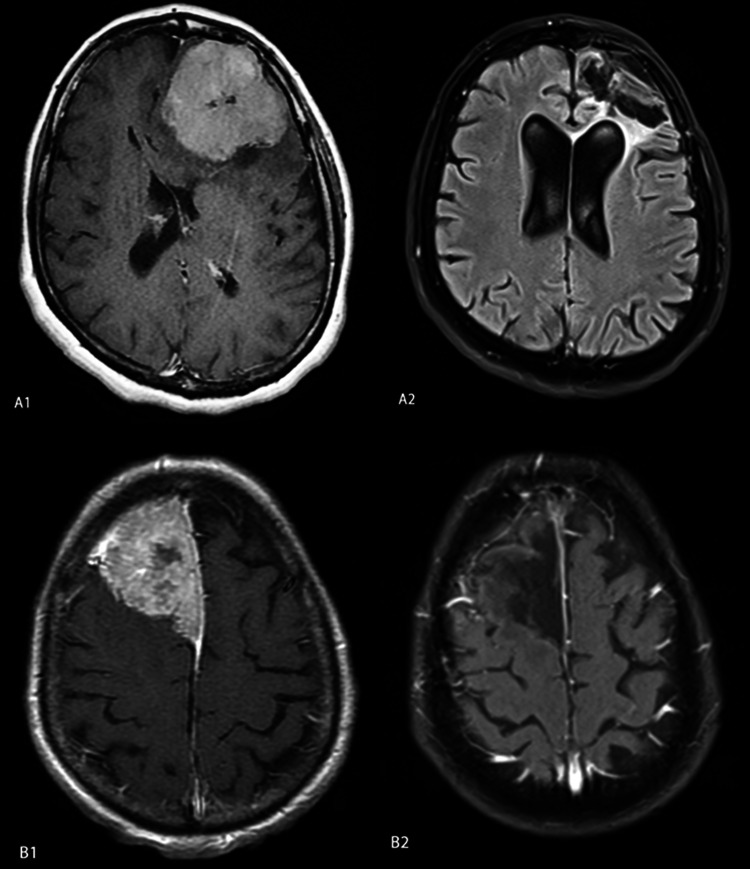
Two case samples for Simpson grade I and grade II tumor resections. A) Grade I B) Grade II resection.

Cranial Computed Tomography and MR imaging were performed on all giant meningiomas. Three patients underwent MR Angiography, six patients underwent MR Venography, and four patients underwent Digital Subtraction Angiography (DSA). Preoperative embolization was done to two of the four patients who underwent DSA. Surgical treatment was performed to all patients. According to the Simpson grading scale, surgical resection rates were considered as grade I in five patients (35.71%), grade II in four patients (28.57%), and grade III in four patients (28.57%). One patient was recorded as grade IV (Table 6 and Figure 2).

**Table 6:** Simpson grading system and surgical results.

Simpson grading system	≥5cm (N=14)	<5cm (N=34)
Grade I- Macroscopically complete removal of the tumor, with excision of its dural attachment, and of any abnormal bone.	5 (35.71%)	23 (67.65%)
Grade II- Macroscopically complete removal of the tumor and of its visible extensions, with endothermy coagulation (usually to the point of charring) of its dural attachment.	4 (28.57%)	8 (23.53%)
Grade III- Macroscopically complete removal of the intradural tumour, without resection or coagulation of its dural attachment, or alternatively, of its extradural extensions, e.g., an invaded sinus or hyperostotic bone.	4 (28.57%)	1 (2.94%)
Grade IV- Partial removal, leaving intradural tumour in situ.	1 (7.14%)	2 (5.88%)

As early complications, cerebrospinal fluid leakage occurred in two patients, infection in one patient, and rhinorrhea in two patients. The findings of patients who developed rhinorrhea improved without requiring surgical intervention. The patient who developed empyema was re-operated, and the infection was cleared. One patient had a major vascular injury during the surgical procedure. He was lost in the fourth postoperative month. This patient's tumor was located in the sphenoid wing, orbital and cavernous sinus invasion (Table 7).

**Table 7:** Postoperative complications.

Postoperative complications	Group 1 N=14(≥5cm)	Group 2 N=34 (<5cm)
Cerebrospinal leakage	2	
Infection	1	4
Rhinorrhea	2	1
Hemiparesis		2
Deep vein thrombosis		1
Major vascular injury during the surgical procedure (Exitus)	1	

According to the histopathological results obtained after surgery, 10 patients have classified as grade I meningioma (71.43%), and four patients were classified as grade II (atypical) meningioma (28.57%). Five of the grade I meningiomas was a transitional type, three were fibrous type, and two were meningothelial type (Table 8).

**Table 8:** Histopathological results of meningiomas in both groups.

Histopathology		Group 1 ≥5cm (N=14)	Group 2 <5cm (N=34)
Grade I	Transitional	5 (35.71%)	8(23.53%)
	Fibrous	3 (21.42%)	11(32.35%)
	Meningothelial	2 (14.29%)	9(26.47%)
	Secretory		2 (5.88%)
Grade II	Atypical	4 (28.57%)	2 (5.88%)
	Rhabdoid		1(2.94%)
	Clear cell		1(2.94%)

None of the first-degree relatives of these patients had meningioma. After obtaining histopathological results, Grade II meningiomas were referred to the Oncology clinic. 

While the average KPS score of the patients was 75.71 before the operation, the average value increased to 85.71 in the postoperative period. The average follow-up time of giant meningiomas was 30.31 months. The minimum follow-up was 10 months, while the maximum was 51 months. One of the operated patients was not included in the follow-up, as he was lost at the fourth postoperative month. In the postoperative short-term follow-up period, the total recurrence rate was found to be 7.69%. While recurrence was not observed in grade I meningiomas, recurrence was observed in one patient (25%) whose histopathological result was atypical meningioma. The patient with recurrence was 67 years old and male. The tumor placement was right parasagittal. The Simpson resection grade was II. Minimal thickening was detected on the sagittal sinus wall on MR imaging one year later.

Meningiomas smaller than 5 cm (Group 2)

The size of the largest meningioma in group 2 was 4.30 cm, and the smallest was 1.50 cm. Of this patient group, 28 were female (82.35%), and six were male (17.65%). While the age range was 31-71, the average age was 50.23 (SD= ± 10.02). When tumors were classified according to their localizations, 18 were in convexity (52.94%), 11 were in the falcine-parasagittal region (32.25%), and two were in an olfactory groove(5.88%). One tumor was in the suprasellar region(2.94%) and one in the tuberculum sella (2.94%). There were also two tumors in one patient in this group (2.94%).

The most common complaints in 34 meningioma cases were headache in 13 (38.23%) and seizure history in eight (23.53%). In addition, visual impairment (5-14.71%), vertigo (3-8.82%), balance disorder (2-5.88%), hearing impairment (2-5.88%), worsening of consciousness (1-2.94%), personality change (1-2.94%), and numbness in the face (1-2.94%) were also detected. There were no complaints in the two patients. In their neurological examinations, a decrease of visual acuity was seen in six patients (17.65%), upper extremity monoparesis in one patient (2.94%), facial paresis in one patient (2.94%), decrease in hearing in one patient (2.94%), and worsening in the level of consciousness in one patient (Glasgow Coma Score 12) (2.94%).

According to Simpson grading scale, surgical resection rates were recorded as grade I in 23 patients (67.65%), grade II in eight patients (23.53%), grade III in one patient (2.94%), and grade IV in two patients (5.88%). Postoperative complications were infection in four patients, hemiparesis in two patients, and rhinorrhea in one patient. Deep vein thrombosis occurred in one patient. Osteomyelitis developed in three of four patients with wound infections. Osteomyelitis developed in one patient three years after surgery. In two patients who developed hemiparesis, muscle strength completely improved after the first month. In one patient, muscle strength improved from 2/5 to 4/5. The complaint of the patient with rhinorrhea improved after repair surgery.

According to the histopathological results obtained after surgery, 30 patients have classified as grade I meningioma (88.23%), and four patients were classified as grade II (11.77%). Of the grade I meningiomas, 11 were fibrous, nine were meningothelial, eight were transitional, and two were secretory-type. Two grade II meningiomas were atypical. One was cordoid and the other clear cell. All meningiomas, the result of which was grade II, were referred to the Oncology clinic.

KPS score average of 34 patients was calculated as 97.35 preoperatively and 96.76 postoperatively. One year later, this average was 99.11. The shortest follow-up was one month and the longest was 49 months. The average follow-up was 27.71 months. During this period, recurrence occurred in one patient in the 11th month. The pathology of the recurrent meningioma was clear cell (grade II).

Statistical assessment

We statistically analyzed our results to evaluate the differences between giant meningiomas and others. When we analyzed a relationship between sex and tumor size, no significant relationship was found as the calculated p was 0.11. (The Spearman Correlation Analysis) Similarly, we investigated whether there is a correlation between age and tumor size. When we subjected the results to the Independent Samples T-test, there was no significant relationship between an age-dependent variable and tumor size (p= 0.13).

When we investigated whether there was a relationship between tumor localization and size, the result we found was not statistically significant (P=0.65) (The Spearman Correlation Analysis). We compared the resection rates between the two groups according to the Simpson grading scale. When the results were subjected to the Spearman Correlation Analysis, we found that the grade I and II rates of the "meningioma smaller than 5 cm" group were significantly higher than the "giant meningioma" group (p=0.02).

We classified and compared both groups according to histopathological results. While the rate of grade I tumors among giant meningiomas (Group 1) was 71.43%, the rate of grade II tumors was 25.87%. In the group with meningiomas less than 5 cm (group 2), the grade I tumor rate was 91.18%, and the grade II tumor rate was 8.82%. These results show that the rates of grade II tumors are higher in giant meningiomas than in the other group. However, there was no significant relationship between tumor size and histopathology (p=0.15) (The Spearman Correlation Analysis).

KPS score averages of the patients were 75.71 for Group 1 and 97.35 for Group 2. Average postoperative KPS score was 85.71 for group 1 and 96.76 for group 2. When we compared the postoperative KPS scores of the groups, the difference was statistically significant (The Mann Whitney U test, p=0.00).

Postoperative early and late complication rates were 42.86% in the first group and 23.53% in the second group. This showed that giant meningiomas had higher rates of surgical complications. When we subjected the results to the Spearman Correlation Analysis, it was not statistically significant (p=0.12).

**Figure 3:**
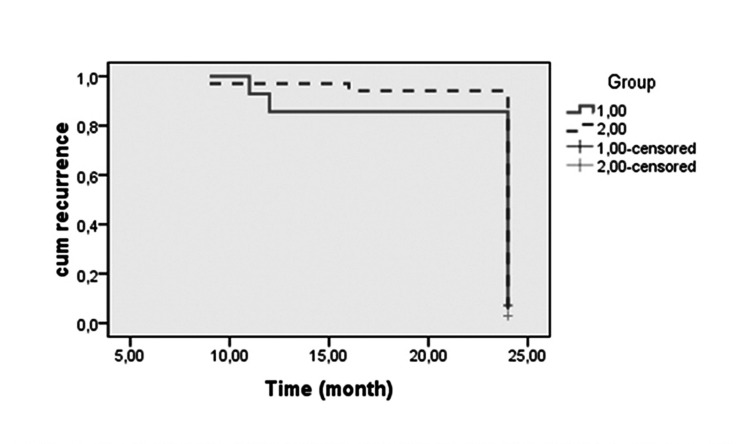
Graph resulting Kaplan Meier survival analysis of recurrences in both groups.

We compared the recurrence rates of both groups with the Kaplan Meier survival analysis graph. When we put the results into the logrank test and calculated the chi-square test statistics, p=0.71. Therefore, there was no significant relationship between tumor size and recurrence rates in our study (Table 9 and Figure 3). 

**Table 9:** Comparison of recurrence rates of both groups through logrank test. (The vector of trend weights is -1, 1. This is the default.)

	Chi-Square	Default	Significance
Logrank (Mantel-Cox)	0.138	1	0.711

## Discussion

We aimed to discuss the results by evaluating and comparing the findings of the two groups of tumors we defined according to their size. The most common localization in Group 1 was the falcine-parasagittal location (35.71%), whereas in Group 2 this was convexity (55.88%). In the study of Ökten et al., who also investigated giant meningiomas, tumors with convexity (24%), falcine-parasagittal (24%), and then olfactory groove (14%) were encountered [15]. According to the incidence, in the study of Özsoy et al. investigating intracranial benign giant meningiomas, tumor placement was listed as convexity, parasagittal and olfactory groove [3]. Also, in the series of Narayan et al., the most common localization was the anterior fossa region (25%). This was followed by the medial sphenoid wing (20%) [2].

The most common complaint in both groups was a headache. In the giant meningioma group, this was followed by nausea and vomiting. In Group 2, the second most common complaint was a history of seizures. It was understandable that the complaint of headache was in the foreground in terms of both dural irritation and increased intracranial pressure. Again, in giant meningiomas, nausea and vomiting followed, attributed to high intracranial pressure. In addition, a history of seizures, which are common in small-sized meningiomas, may be a sign of cortical irritation rather than mass effect.

Magill et al. and Ressel et al. found a statistically significant relationship between tumor size and histopathological results. In both, it was concluded that the rate of grade II tumors was higher in larger tumors [8,9]. In our study, the rate of grade II tumors was higher in giant meningiomas. However, the relationship was not found to be significant. The comparative statistical analyzes we documented in the "Results" section show that the points in the items we highlight below are important: A) Resection rates are significantly lower in group 1 with giant meningiomas. B) In group 1 with giant meningiomas, KPS scores are significantly lower than group II. C) When statistical comparisons were made according to sex, age, histopathological results, complications, and recurrence rates, no significant differences were observed. D) The term "Giant Meningioma" is a type of distinction that is frequently made in the literature. However, our study shows that giant meningiomas do not make any further difference other than surgical results (Table 10).

**Table 10:** Comparison of two groups of meningioma according to “P” values.

Variables	P value	Significance
Sex	0.11	No
Age	0.13	No
Localization	0.65	No
Resection rate	0.02	Significant
Histopathological result	0.15	No
Karnofsky Performance score	0.00	Significant
Complication	0.12	No
Recurrence rate	0.71	No

Although there are exceptions, there is a consensus that surgical resection of the tumor is the first option in treating meningioma. Wide surgical resection, histopathological grading, patient age, tumor size, and localization can be counted as prognostic factors for survival of the disease [16-22]. Some studies indicate that the recurrence rate of parasagittal and falcine meningiomas is higher than others. This is due to the difficulty of radical resection. In addition, it is stated that the risk of recurrence is higher in African Americans [23,24]. In cases such as advanced age, cardiovascular problems, tumor placement, and asymptomatic cases, the first option may not be surgical [25]. Nevertheless, in giant meningiomas, surgical resection can be the only viable option as the mass effect progressively deteriorates the patient's neurological condition.

Together with this priority, our study showed that the size of the tumor affected the resection rates, the risk of perioperative complications, and the general condition of the patients before and after surgery.

Limitations of the study

Having a more extensive patient series would have made our results stronger. However, this study had to be limited to the current number of cases due to its retrospective nature. Two patients with multiple meningiomas in both groups were genetically investigated. No significant findings were found as a result of genetic examinations of the patients with multiple meningiomas. In our opinion, genetic examination of all patients would strengthen our study. In addition, we suspect that giant meningiomas may be more likely among the population that cannot reach health checkpoints. We think that this subject should be a separate research topic.

## Conclusions

When the giant meningiomas are compared with smaller tumors, the differences are those that the neurosurgeon must compensate directly. These differences are lower resection rates, higher complication rates, and high morbidity and mortality risk. The size of the tumor may affect the general condition of the patients primarily in operation. Prolonged surgery can cause systemic medical and anesthetic problems. Larger tumors also require larger skin flaps and larger craniotomy, leading to the ground for increased postoperative morbidity. In addition, the larger tumor size poses a more significant threat to the intracranial neural and vascular structures both on its own and during surgery. Because of all this, methods to minimize the risks caused by size in surgical planning should be considered. Preoperative preparations should also be made considering these risk factors. Furthermore, a high resection rate and also low morbidity should be targeted during surgery.
